# Effect of a Combination of Lysolecithin, Synthetic Emulsifier and Monoglycerides on the Apparent Ileal Digestibility, Metabolizable Energy and Growth Performance of Growing Pigs

**DOI:** 10.3390/ani13010088

**Published:** 2022-12-26

**Authors:** La Van Kinh, Bindhu L. Vasanthakumari, C. Sugumar, Huyen La Thi Thanh, Nguyen Van Thanh, Alexandra L. Wealleans, Le Duc Ngoan, Nguyen Vu Thuy Hong Loan

**Affiliations:** 1 Faculty of Veterinary and Animal Science, HUTECH University, 475A Dien Bien Phu, Ho Chi Minh City 717000, Vietnam; 2 Kemin Industries, Des Moines, IA 50317, USA; 3 Kemin Industries (Asia) Pte Ltd., 12 Senoko Dr, Singapore 758200, Singapore; 4 Institute of Animal Sciences for Southern Vietnam, Di An Town, Binh Duong City 82000, Vietnam; 5 Kemin Animal Nutrition and Health, Kemin Europa N.V., Toekomstlaan 42, 2200 Herentals, Belgium; 6 Faculty of Animal Sciences and Veterinary Medicine, Hue University of Agriculture and Forestry, Hue University, 102 Phùng Hưng, Hue City 530000, Vietnam

**Keywords:** apparent ileal digestibility, growth performance, lysophospholipids, metabolizable energy, pigs

## Abstract

**Simple Summary:**

As the primary source of dietary energy, improving fat digestibility can have positive impacts on performance and profitability in pig production. Use of lysolecithin to improve fat and other nutrient digestibility may be able to increase the growth rate and efficiency of growing pigs. In the present study, a combination of lysolecithin, synthetic emulsifiers and monoglycerides was able to significantly increase nutrient and amino acid digestibility in growing pigs, driving significant increases in growth, efficiency and the economics of production.

**Abstract:**

Two studies were conducted to determine the impact of an absorption enhancer containing a combination of lysophospholipids, monoglycerides and synthetic emulsifiers (LEX) on apparent ileal digestibility, metabolizable energy (ME), and growth performance of growing pigs. In the digestibility study, 12 male crossbred [Duroc x (Large White x Landrace)] pigs with an initial body weight (BW) of 30 kg were randomly allocated to two dietary treatments: (1) a positive control (PC) receiving standard diets formulated to 3100 kcal ME/kg, and (2) a negative control formulated with −100 kcal ME/kg and −2.5% AA content vs. PC and supplemented with LEX at 500 g/t. Apparent ileal digestibility of essential AA was significantly increased for lysine, methionine, threonine, histidine, isoleucine and phenylalanine in the LEX treatment (*p* < 0.05). Average uplift in AA digestibility as a percent of the PC was +1.88%; with greatest improvement for histidine (+4.22%). LEX supplementation effectively compensated energy deficiency of low-density diet and provided additional ME improvement compared to the PC diet (*p* < 0.05). A second study was conducted to evaluate LEX dose response on the growth performance of pigs fed a low nutrient density diet. Total 450 cross-bred pigs (240 males (castrated) and 210 females) [Duroc x (Large White x Landrace)] with an average initial BW of 7.19 kg were randomly allocated into 4 dietary treatments: (1) a positive control (PC) formulated with standard specifications, (2) a negative control formulated with −100 kcal ME/kg and −2.5% AA content vs. the PC (NC), (3) NC + LEX at 250 g/t (NC + 250) and (4) NC + LEX at 500 g/t (NC + 500). Results showed significant improvement with LEX supplementation on the final BW, average daily gain (ADG) and feed conversion ratio (FCR) of pigs of >50 kg body weight. Across the study, NC + 500 significantly increased ADG vs. PC and NC, and significantly reduced FCR compared to all other treatments. FCR of negative control diets improved by 9 and 15 points with the supplementation of 250 g/t and 500 g/t of LEX, respectively (*p* < 0.05). FCR in the NC + 250 diet was statistically similar vs. PC, which was significantly reduced compared to the NC. Taken together, these studies demonstrate that the addition of an absorption enhancer containing a combination of lysophospholipids, monoglycerides and synthetic emulsifiers can improve growth performance in growing pigs, driven by increased nutrient digestibility and retention.

## 1. Introduction

Efficient fat utilization has great economic significance in maintaining profitable pig production since fat is both an important and expensive feed ingredient [1]. The practical value of fats and oils in swine diets is related to their digestible energy value [2], and studies have demonstrated that adding fat to pig diets improved daily weight gain and feed efficiency [3,4]. Though fat digestion takes place in an aqueous environment, lipids themselves are not water soluble; therefore, one strategy to improve the utilization and retention of energy and fatty acids from dietary fat is the use of absorption enhancers which can help improve the different stages of fat digestion—emulsion, hydrolysis, and absorption. 

Lysophospholipids can improve the emulsification of feed fats, advance mixed micelle formation, and enhance the absorption of nutrients by increased cell membrane permeability [5,6,7,8]. Zhang et al. [6] reported that lysophospholipids act together with bile salts as an emulsifier within the first stage of lipid digestion. Lysophospholipids can participate in the formation of mixed micelles, and they are known to improve the absorption of lipids and other nutrients [8,9], including saturated lipids that normally would have lower potential for micelle formation [8,10]. 

Previous research has shown the potential of combining lysophospholipids with other active ingredients such as monoglycerides and synthetic surfactants in improving the absorption of nutrients [11]. In poultry, the combination has been shown to improve growth performance and efficiency [10,12,13,14] as well as improving gut structure and function [15,16]. However, there is a paucity of published information on the use of the combination of lysophospholipids, monoglycerides and synthetic surfactants in growing pigs. Therefore, the present study aimed to evaluate the effect of a combination of lysophospholipids, monoglycerides and synthetic surfactants on nutrient digestibility, metabolizable energy and growth performance in growing pigs.

## 2. Materials and Methods

Both studies were carried out at the Research Station of Institute of Animal Sciences for Southern Vietnam in Ho Chi Minh City. All experimental procedures were in line with commercial practices, compliant with all local animal welfare legislation, and approved by the Institute of Animal Sciences for Southern Vietnam in Ho Chi Minh City.

### 2.1. Digestibility Experiment

#### 2.1.1. Experimental Design 

A total of 12 weaned piglets (30 kg live weight) were allocated to two treatments and housed in individual metabolism cages. The positive control (PC) diet was a commercial feed containing 3100 kcal ME/kg, formulated to meet all nutrient requirements. The remaining diet was reformulated to −100 kcal ME and −2.5% amino acid compared to the positive control and supplemented with 500 g/t of a combination of lysophospholipids, monoglycerides and synthetic emulsifiers (NC + 500, Lysoforte^®^ Extend, Kemin Industries, Des Moines, IA, USA (LEX)). Ingredient and nutrient composition of the experimental diets is shown in Table 1. Animals were individually kept in metabolism cages for 12 days, with 7 days adaptation and 5 days for faeces collection. At day 13, immediately after the collection period, all animals were slaughtered for ileal digesta collection following the procedure of Moughan et al. [17].

#### 2.1.2. Chemical Analysis

Chromium oxide (Cr_2_O_3_) was added at 0.2% (DM basis) to diets as an indigestible marker. All feed refusals and faeces were collected daily and weighed and stored at −20 °C prior to subsampling. At the end of collection period, subsamples (equivalent to ca. 700 g DM) were taken after carefully mixing 5-day collected feed refusals and faeces. 

Digesta samples were weighed, homogenized and frozen at −20 °C. Sub-samples of feed refusals and faeces were dried at 60 °C; and digesta samples were freeze-dried prior to chemical analyses. Samples were subsequently ground and analyzed in duplicate. Chemical analyses including amino acid analysis were run at Institute of Animal Sciences in Ho Chi Minh City according to AOAC methods (2002 and 2006) [18,19]. CP was determined using copper catalyst Kjeldahl method. Gross energy (GE) was analysed by bomb calorimeter [20] following the Operating instruction manual (Parr 6100 Compensated Jacket Calorimeter 2013). Amino acid contents were determined using AA-analyzer (L-8900, HITACHI. Japan). The apparent ileal digestibility of protein and amino acid at each sampling site was calculated using the indicator technique [21] according to the equation:

ADD = 100 × [(1 − (DCf/DCd × Id/If)]

where ADD is the apparent digestibility (ileal or total tract) of a dietary nutrient/component; DCf the dietary nutrient/component concentration in ileal digesta or faeces (g/kg); DCd the dietary nutrient/component concentration in the diet (g/kg); Id the Cr_2_O_3_ concentration in the diet (g/kg); If the Cr_2_O_3_ concentration in ileal digesta or faeces (g/kg).

The ME of the diets was calculated as follows [22]:

DE = Digestibility coefficient × GE

ME = DE × (1.003 − 0.0021×%CP)

### 2.2. Growth Trial

#### 2.2.1. Animals, Experimental Design, and Diets

Four hundred and fifty pigs ([Duroc × (Large white × Landrace)]) with an average initial weight of 7.19 kg (SD = 0.2) and of similar age of 28 days were randomly allocated according to gender to pens. Fifteen pigs (8 males and 7 females) per pen were randomly allocated to one of the four dietary treatments with different levels of supplements as follows: (1) a positive control diet formulated to meet commercial specifications (PC), (2) a negative control (NC) formulated with 100 kcal ME/kg less ME and 2.5% lower amino acid content compared to PC, (3) NC supplemented with 250 g/t of LEX (NC + 250), and (4) NC supplemented with 500 g/t of LEX (NC + 500).

Before the study, from weaning at 28 days of age, all pigs were fed a common corn–soybean meal-based diet formulated to meet the nutritional requirements suggested by National Research Council (NRC, 2012). Ingredient and nutrient composition of the experimental diets is shown in Table 1. Throughout the growing period, pigs were fed the diets depending on growing stage (28–56 days, 56–112 days, and 112–168 days of age). The diets used in the study were based on corn, wheat and SBM, with additional fish meal, rice bran and wheat bran. PC diets were formulated according to commercially relevant specifications, and the NC diets were reduced in energy (100 kcal ME/kg) and amino acids (2.5%), as shown in Table 1. On top of the NC diet, a combination of lysophospholipids, monoglycerides and synthetic emulsifiers (LEX) was supplemented at either 250 or 500 g/t. The combination of lysophospholipids, monoglycerides and synthetic emulsifiers used in the study was LYSOFORTE^®^ EXTEND (Kemin Industries Asia, Singapore). All pigs were provided with ad libitum access to feed and water through a self-feeder and nipple drinker, respectively. 

#### 2.2.2. Performance Measurements and Economic Calculations

Pigs were housed in pens and fed the experimental diets for 140 days. Live body weight (BW) was recorded at initial (28 days of age), 56, 112 and 168 days of age. Feed intake was monitored daily to calculate average daily feed intake (ADFI) per phase and overall. Feed refusals (from the previous day) were collected every morning before daily feeding. Collected feed was then dried to attain a moisture content of 13% and weighed. Average daily weight gain (ADG) and feed conversion ratio (FCR) were then calculated from overall bodyweight gain and total feed intake. At the end of the experiment, backfat thickness at P2 was measured by Aloka machine.

Using the method of Wealleans et al. [23], performance results and diet costs were used to assess the cost–benefit of reformulation and LEX supplementation. Briefly, the return over expenses compared to both PC and NC diets was determined for the NC + 250 and NC + 500 treatments by subtracting the costs from the gross revenue generated by liveweight of the pigs at the end of the growth period. Prices used were representative of Q4 2020.

#### 2.2.3. Microbial Analysis of Faeces

Total bacterial count and coliform counts were determined using standard methods [24,25]. Total aerobic bacteria: total aerobic bacteria in faecal samples formed colonies in an oxygen rich environment. The bacterial colonies were then counted by pouring Petri-dish method using Nutrion Agar (NA) at 37 °C/72 h, pH 7.0 ± 0.2. Coliforms count: Coliforms in faecal sample were counted using Petri-dish method with Violet Red Bile agar (VRB) at 45 °C/24 h and was confirmed in E. coli medium broth (EC) at 44 °C/24–48 h.

### 2.3. Statistical Analysis

Data were analysed by ANOVA using Minitab Statistical Software version 16.2.0 (2010). Differences between means were tested by using Tukey’s Honest Standard Difference. An analysis of variance was done according to the following model:

Yij = μ + Ti + eij

where Y is a dependent variable, μ is the overall mean, Ti is the treatment effect (i = 1, 2, 3, ...) and eij is the random error. *p* < 0.05 was considered statistically significant; *p* < 0.1 was considered to indicate a near-significant trend.

## 3. Results

### 3.1. Digestibility Experiment

The intake of dry matter and crude protein did not differ significantly across the treatments (*p* > 0.05); whereas the ME intake was significantly lower in the PC compared to NC + 500 (*p* < 0.05) due to higher ME concentration (*p* < 0.05) (Table 2). However, LEX supplementation to the low-density diet facilitated compensation of nutrient deficit and extra improvement in ME utilization compared to the control (*p* < 0.05).

LEX supplementation improved the ileal digestibility of crude protein and essential amino acids, as shown in Table 3. The digestibility results showed that the pigs fed the NC + 500 diet experienced a significant increase in the digestibility of histidine, threonine, tyrosine, methionine and lysine compared to the PC and there was a statistical trend towards increased isoleucine digestibility (Table 3). Average uplift in amino acid digestibility following LEX supplementation as a percent of the positive control was +1.88%; the greatest improvement was seen for histidine which saw a 4.22% improvement. Lysine, the first limiting amino acid, saw a 3.5% improvement in availability following LEX supplementation.

### 3.2. Growth Study

The results of BW, DMI, ADG and FCR in three growth stages are shown in Table 4. During the first stage of the study (day 28–56) there was no significant difference in BW, ADG, FI or FCR, despite some relative differences in growth and FCR. This may be due to the large variation seen in the growth and feed intake of newly weaned pigs, which is driven by the dual stressors of weaning (new feed source and method of feed intake) and the mixing of litters into larger social groups.

There was a significant difference in the BW of pigs at 112 and 168 days across the different treatments (*p* < 0.05). Reduction in ME and amino acid content resulted in lower growth performance of NC compared to the PC (*p* < 0.05), while LEX supplementation on top of NC diets resulted in significant improvement in body weight, and average daily gain (ADG) as the pigs grew older (112 days and beyond). Final BW of pigs fed NC + 500 was higher than that of both the NC and PC (*p* < 0.05). Daily feed intake in pigs across all treatments were not significantly different (*p* > 0.05). Feed conversion ratio (FCR) showed differences across treatments at 56–112 and 112–168 age days. At 56–112 days, NC + 500 resulted in a 14 point improvement in FCR compared to the NC group during both the 56th–112th day period and 112th–168th day period (*p* < 0.05).

Growth performance for the entire study period and the backfat thickness at slaughtered final weight are presented in Table 5. Reduction in metabolizable energy and amino acid content had significant impact on growth performance of pigs during the trial period as evident from the lower average daily gain and higher FCR of NC group (*p* < 0.05). Supplementation of LEX facilitated improvement in growth performance with the most significant effect produced by LEX at 500 g/t. Feed conversion ratio (FCR) of negative control diets was improved by 0.09 and 0.15 with the supplementation of 250 g/t and 500 g/t of LEX, respectively (*p* < 0.05). There was no significant difference in backfat thickness between treatments on day 168. Likewise, there was no significant difference in feed intake between treatments throughout the study.

Reformulation of growing pig diets and supplementation with LEX also demonstrated feed cost savings compared with the PC. NC + 250 resulted feed cost savings of 10.76 $/t for the starter diet, 9.23 $/t in the grower and 4.71 $ per tonne in the finisher compared to the positive control (Table 6). Likewise, the NC with 500 g/t LEX produced a feed cost saving of 9.31 $/t in the starter, 7.78 $/t in the grower and 3.26 $/t in the finisher. When the increased feed intakes are considered, this resulted in NC + 250 and NC + 500 demonstrating total feed cost savings compared to the NC of 0.97 $ and 0.61 $ per pig produced, respectively.

The average profit margin per pig was also substantially increased for both NC + 250 and NC + 500 compared to the positive control, with pigs fed the higher dose of LEX achieving increased profits of almost 8 USD per pig more than the positive control. When reformulation and improved performance following supplementation with LEX are considered, the changes resulted in improved profit per tonne of feed produced of 15.33 and 26.22 USD for 250 and 500 g/t, respectively.

### 3.3. Microbial Analysis

Microbial analysis of faecal samples showed the highest total bacteria count and coliform count for samples collected from pigs fed with the commercially relevant PC diet (Table 7). There was a decreasing trend in total bacteria and coliforms count when pigs were fed low nutrient density diet (negative control diet) with the LEX supplementation indicating some benefits in decreasing the counts further, though not significant (*p* > 0.05). It appeared that supplementing LEX in pig diets could help to improve digestibility a enhance useful microbial flora of pig intestine.

## 4. Discussion

Several factors limit the digestion and absorption of lipids including the age of animals, type of fat, and emulsification of lipids within the gastrointestinal tract [8,26,27]. Generally, the digestibility of lipids (particularly when animal fat sources are used) in young pigs is low and increases with age [28,29,30]. The digestibility of lipid sources is impacted by the chemical composition of the lipids and the quality of fat sources, including moisture content, impurities, unsaponifiables, and oxidative stability [31,32]. The ability of lipids to form micelles with bile salts in the lumen of the intestine is directly related to the digestibility of the lipid [27].

The beneficial effect of lysophospholipids on all three steps of the fat digestion process (emulsification, hydrolysis and absorption) has been extensively researched [9]. Wealleans et al. [10] showed that the addition of lysophospholipids at levels of 125 g/t and above to broiler diets consistently improved feed efficiency across a range of basal dietary ingredients and fat sources. The addition of selected quantities of synthetic emulsifier and monoglycerides to lysophospholipids as a mixture has been reported to improve lipid emulsification and hydrolysis, respectively resulting in improved broiler performance [12,13]. The current study was performed to evaluate the effect of this combination of lysophospholipids,monoglycerides and synthetic surfactant LEX in improving the nutrient digestibility, metabolizable energy and growth performance of growing pigs.

The major finding from present study is the ability of LEX to improve the utilisation of metabolizable energy, dietary nutrients and amino acids by growing pigs. There was a significant increase in the digestibility of essential amino acids (lysine, isoleucine and phenylalanine) with the use of LEX (*p* < 0.05). Previous studies conducted on weaning and lactating pigs have shown similar results: Jones et al. [5] observed an increase in fat digestibility when lecithin or lysophospholipids were added to nursery diets containing soybean oil or tallow, but not in diets containing lard. Zhao et al. [33] reported that the addition of lysophospholipids improved apparent total tract digestibility of DM, N, GE, and crude fat of weaning pigs, while supplementing with lysophospholipids improved nutrient digestibility of lactating sows [34]. The nutrient digestibility improvements observed in the present study may be attributed to the superior emulsification properties of LEX. The incorporation rate of lipids into micelles is limited by the extent of digestion within the small intestine and stomach. Studies show that the addition of selected quantities of synthetic emulsifier and monoglycerides to lysophospholipids and applying it as a mixture (LEX) result in improved in vitro lipid emulsification and hydrolysis [2,11]. An in vitro absorption study using Caco-2 cells showed a significant improvement in free fatty acids by more than 75% [11]. Broiler studies have established the efficacy of the lysophospholipids-monoglyceride-synthetic surfactant system in improving the energy utilization and slaughter yield [11,12,14]. Therefore, the greater digestibility of nutrients in the presence of LEX in the current study could be attributed to the efficient emulsification and formation of stable mixed micelles in the gut environment.

The improved emulsification of fats could not only enhance the digestion of the lipids but also of other nutrients. For example, Honda et al. [35] found that fats incorporated in the feed matrix could cover other nutrients, lowering their digestion. In fact, a greater effect on the non-fat fraction of the feed was also observed in pigs [36,37]. Furthermore, the reason for higher nutrient bioavailability by LEX in the present study may be attributed to the ability of lysophospholipids to enhance the uptake of nutrients across the enterocyte membrane [38] by inducing alterations in protein channel formation and increasing ion exchanges [39,40]. The incorporation of lysophospholipids in membranes induces local curvature of bilayers [41,42], increasing fluidity and permeability of the membrane. Brautigan et al. [16] observed increased expression of collagen coding genes, along with increased villi collagen cross-linkages and height with the use of lysophospholipids. Several further studies have confirmed positive effects of lysophospholipids on the intestinal morphology in pigs and poultry [43,44].

Lysophospholipids supplementation has been reported to have beneficial effects on gut microbiota, integrity and gene expression. Polycarpo et al. [45] found reduced levels of Gram-positive cocci in the jejunum, which has been explained as a combination of direct disruption of the bacterial cell membrane [46] and reduced fat available for bacterial growth in the lumen. Similarly, the present study results indicate a decreasing trend in total bacteria and coliform counts when pigs were fed diets supplemented with 250 g/t LEX. Further studies are needed to explain the exact mechanism between lysophospholipid supplementation and gut microbiota.

The present study showed that the addition of LEX significantly increased the performance of growing pigs fed lower nutrient dense diets, which reinforces the findings of previous studies that reported beneficial effect of lysophospholipid supplementation on the performance of weaning pigs [33]. However, the effect of lysophospholipids supplementation on swine performance are inconsistent: Zhao et al. [34] observed that piglets’ performance was not affected by lysophospholipid supplementation. The reasons for these inconsistent results may be attributable to the age of pigs, the fat sources, variability in feed ingredients and the feed reformulation strategy used [31] and requires further investigation.

## 5. Conclusions

Lysophospholipids in combination with monoglycerides and synthetic surfactants (LEX) improved the nutrient digestibility and growth performance of pigs fed low nutrient dense diets. Supplementation of LEX could be used to improve growth performance and profitability of commercial pig production.

## Figures and Tables

**Table 1:** Composition and nutritional levels of the experimental diets.

Raw Materials, %	Starter 28–56 Days		Grower 56–112 Days		Finisher 112–168 Days	
	PC	NC	PC	NC	PC	NC
Corn	45.73	41.43	44.54	41.2	35.84	29.38
Wheat	15	15	20	20	25	25
Full fat soybean	6	6	-	-	-	-
Soybean meal (47% CP)	12.7	10.5	19.77	17.44	17.07	14.46
Soytide (Fermented soy)	5	5	-	-	-	
Whey powder (11% CP)	3	3	-	-	-	-
Fish meal (60% CP)	3	3	2	2	-	-
Rice bran	-	-	10	6.27	15	15
Wheat bran	-	7.56	-	10	3.78	12.89
Dicalcium phosphate(DCP), 17% P	1.02	0.98	0.98	0.91	0.86	0.73
Limestone	1.07	1.11	1.06	1.12	1.48	1.56
Lactose	5	5		-	-	-
L-Lysine 98%	0.37	0.37	0.26	0.27	0.17	0.18
DL-Methionine	0.13	0.13	0.04	0.03	0.01	0.01
L-Threonine	0.18	0.18	0.08	0.08	0.06	0.07
L-Tryptophan	0.06	0.06	-	-	-	-
Soybean oil	1.05	-	0.58	-	-	-
Salt	0.32	0.31	0.32	0.31	0.36	0.35
Premix	0.37	0.37	0.37	0.37	0.37	0.37
Total	100	100	100	100	100	100
Calculated Chemical Composition, %						
DM	90.54	90.34	88	87.96	87.99	88.05
ME (kcal/kg)	3400	3300	3200	3100	3100	3000
Crude protein	19	18.35	17.5	17.06	16	15.6
Crude fat	4.81	4.62	4.9	4.36	4.23	4.63
Crude fibre	2.85	3.17	3.66	4.12	4.64	5
Ca	0.9	0.9	0.8	0.8	0.8	0.8
P	0.4	0.4	0.36	0.36	0.3	0.3
Lysine	1.35	1.32	1.1	1.07	0.9	0.88
Methionine	0.43	0.42	0.33	0.32	0.28	0.27
M + C	0.76	0.74	0.65	0.63	0.58	0.57
Threonine	0.87	0.85	0.72	0.7	0.64	0.62
Tryptophan	0.25	0.24	0.21	0.2	0.19	0.19

**Table 2:** Effect of supplementation with a combination of lysophospholipids, monoglycerides and synthetic emulsifiers (LEX) at 500 g/t on the intake of dry matter, crude protein, metabolizable energy and gross energy and the metabolizable energy of growing pigs.

Parameter	PC	NC + 500	SEM	*p*-Value
Dry Matter Intake (g/day)	803.5	817.6	10.12	0.512
Crude Protein Intake (g/day)	140.7	139.3	1.82	0.696
Gross Energy Intake (kcal/day)	3180	3178	38.93	0.980
ME (kcal/kg feed)	3021 ^b^	3207 ^a^	46.09	0.037
ME Intake (kcal/day)	2427 ^b^	2622 ^a^	50.28	0.046

Treatment effects relative to the control group (%). ^a,b^ Means with different letters in the same row indicate significant differences at *p* < 0.05.

**Table 3:** Effect of supplementation with a combination of lysophospholipids, monoglycerides and synthetic emulsifiers (LEX) at 500 g/t on apparent ileal digestibility of crude protein and amino acids (%) in growing pigs.

Parameter	PC	NC + 500	SEM	*p*-Value
CP	84.36	85.11	0.310	0.238
Lysine	82.39 ^b^	85.27 ^a^	0.730	0.040
Methionine	84.6 ^b^	87.3 ^a^	0.680	0.041
Tryptophan	90.39	92.34	0.736	0.155
Threonine	82.51 ^b^	85.8 ^a^	0.610	0.001
Aspartic acid	80.51	81.53	0.571	0.401
Serine	79.65	80.47	0.494	0.428
Glutamic acid	85.80	85.88	0.286	0.902
Glycine	81.31	82.46	0.542	0.309
Histidine	84.19 ^b^	87.74 ^a^	0.680	0.002
Arginine	83.93	84.56	0.353	0.403
Alanine	84.48	85.45	0.584	0.432
Proline	81.78	82.21	0.359	0.556
Cystine	86.30	88.2	1.679	0.596
Tyrosine	81.70	83.29	0.437	0.067
Valine	82.52	83.68	0.360	0.107
Isoleucine	80.62	82.22	0.479	0.096
Leucine	84.48	85.26	0.244	0.116
Phenylalanine	81.08	82.81	0.575	0.139

Treatment effects relative to control group (%). ^a,b^ Means with different letters in the same row indicate significant differences at *p* < 0.05.

**Table 4:** Effect of supplementation with a combination of lysophospholipids, monoglycerides and synthetic emulsifiers (LEX) on the growth performance of pigs at 250 and 500 g/t ^1^.

Parameter	PC	NC	NC + 250	NC + 500	SEM	*p*-Value
Body weight (kg)						
d28	7.21	7.14	7.20	7.14	0.055	0.842
d56	17.99	17.58	17.97	18.36	0.33	0.250
d112	53.81 ^b^	52.19 ^c^	54.12 ^ab^	55.20 ^a^	0.30	<0.001
d168	97.41 ^b^	94.93 ^c^	97.88 ^ab^	99.97 ^a^	0.43	<0.001
Average daily gain (g)						
d28–56	359	347	358	373	4.05	0.170
d56–112	639 ^a^	618 ^b^	645 ^a^	657 ^a^	4.40	0.005
d112–168	778 ^ab^	754 ^b^	781 ^ab^	799 ^ab^	4.82	0.056
Dry matter intake (g/day)						
d28–56	589	590	602	597	2.35	0.134
d56–112	1560	1555	1570	1562	4.01	0.617
d112–168	2444	2436	2430	2435	4.32	0.702
Feed conversion ratios (FCR)						
d28–56	1.64	1.70	1.68	1.60	0.018	0.274
d56–112	2.44 ^ab^	2.52 ^a^	2.43 ^ab^	2.38 ^b^	0.017	0.020
d112–168	3.14 ^ab^	3.19 ^a^	3.11 ^ab^	3.05 ^b^	0.019	0.046

^a,b,c^ Means with different letters in the same row indicate significant differences at *p* < 0.05. ^1^ Diets were fed in three phases: starter 28–56 days, grower 56–112 days, finisher 112–168 days.

**Table 5:** Effect of supplementation with a combination of lysophospholipids, monoglycerides and synthetic emulsifiers (LEX) on the overall growth performance of growing pigs (28–168 days).

Parameter	PC	NC	NC + 250	NC + 500	SEM	P
Initial BW (kg)	7.21	7.14	7.20	7.14	0.055	0.842
Final BW (kg)	97.41 ^b^	94.93 ^c^	97.88 ^ab^	99.97 ^a^	0.43	<0.001
ADG (g)	709 ^ab^	686 ^b^	713 ^ab^	728 ^a^	3.44	<0.001
DMI (g/day)	2003	1995	2000	1998	2.72	0.872
FCR	2.82 ^b^	2.89 ^a^	2.80 ^b^	2.74 ^c^	0.013	<0.001
Back fat thickness (mm)	12.60	12.17	11.80	11.32	0.20	0.122

^a,b,c^ Means with different letters in the same row indicate significant differences at *p* < 0.05.

**Table 6:** Economic calculations in US dollars ($ USD) for growing-finishing pigs fed either a nutritionally adequate diet or a diet reduced in energy and supplemented with a combination of lysophospholipids, monoglycerides and synthetic emulsifiers (LEX) at 250 or 500 g/t.

Parameter	PC	NC	NC + 250	NC + 500
Feed Costs/tonne, $ USD				
Day 28–56	345.27	333.059	334.51	335.96
Day 56–112	300.07	289.39	290.84	292.29
Day 112–168	261.44	255.28	256.73	258.18
Total BWG (kg)	90.20	87.78	90.68	92.83
Total FI (kg)	305.87	302.83	304.41	304.91
FCR	3.14	3.19	3.11	3.05
Income per pig @ 1.88$/kg live weight	183.48	178.81	184.37	188.30
Average total feed cost/pig ($)	105.61	100.86	101.83	102.44
Average feed cost savings/pig ($)	-	4.75	−0.97	−0.61
Average profit/pig ($)	77.87	77.95	82.54	85.87
Net benefit per pig ($)		0.08	4.67	7.99
Net profit/t of feed ($)		0.26	15.33	26.22

**Table 7:** Effect of supplementation with a combination of lysophospholipids, monoglycerides and synthetic emulsifiers (LEX) at 250 and 500 g/t on microbial counts in the faeces of growing pigs.

Parameter			cfu/g		
	Treatment	N	Average	Min	Max
Total bacterial count	PC	6	9.4 × 10^7^	3.35 × 10^7^	14.7 × 10^7^
	NC	6	7.8 × 10^7^	4.38 × 10^6^	151 × 10^7^
	NC + 250	6	5.03 × 10^7^	1.28 × 10^7^	13.3 × 10^7^
	NC + 500	6	3.8 × 10^7^	1.37 × 10^6^	6.57 × 10^7^
Coliforms count	PC	6	8.02 × 10^9^	1.37 × 10^9^	4.88 × 10^10^
	NC	6	6.8 × 10^9^	24.3 × 10^8^	9.86 × 10^9^
	NC + 250	6	5.13 × 10^9^	1.69 × 10^9^	2.17 × 10^10^
	NC + 500	6	4.03 × 10^9^	4.63 × 10^8^	7.86 × 10^9^

## Data Availability

Not applicable.
